# Social support and health-related quality of life among the oldest old — longitudinal evidence from the multicenter prospective AgeCoDe-AgeQualiDe study

**DOI:** 10.1007/s11136-021-03070-2

**Published:** 2021-12-22

**Authors:** André Hajek, Christian Brettschneider, Tina Mallon, Hanna Kaduszkiewicz, Anke Oey, Birgitt Wiese, Siegfried Weyerer, Jochen Werle, Michael Pentzek, Angela Fuchs, Ines Conrad, Melanie Luppa, Dagmar Weeg, Edelgard Mösch, Luca Kleineidam, Michael Wagner, Martin Scherer, Wolfgang Maier, Steffi G. Riedel-Heller, Hans-Helmut König

**Affiliations:** 1 Department of Health Economics and Health Services Research, Hamburg Center for Health Economics, University Medical Center Hamburg-Eppendorf, Hamburg, Germany; 2 Department of Primary Medical Care, Center for Psychosocial Medicine, University Medical Center Hamburg-Eppendorf, Hamburg, Germany; 3 Institute of General Practice, Faculty of Medicine, Kiel University, Kiel, Germany; 4 Institute of General Practice, Hannover Medical School, Hannover, Germany; 5 Medical Faculty Mannheim, Central Institute of Mental Health, Heidelberg University, Mannheim, Germany; 6 Institute of General Practice, Medical Faculty, Heinrich-Heine-University Düsseldorf, Düsseldorf, Germany; 7 Institute of Social Medicine, Occupational Health and Public Health, University of Leipzig, Leipzig, Germany; 8 Department of Psychiatry, Technical University of Munich, Munich, Germany; 9 Department of Neurodegenerative Diseases and Geriatric Psychiatry, University Hospital Bonn, Bonn, Germany; 10 German Center for Neurodegenerative Diseases (DZNE), Bonn, Germany

**Keywords:** EQ VAS, EQ-5D, Health-related quality of life, Oldest old, Social isolation, Social support

## Abstract

**Purpose:**

The aim of this study was to examine the longitudinal within-association between social support and health-related quality of life among the oldest old.

**Methods:**

Longitudinal data (follow-up waves 7 to 9) were used from the multicenter prospective cohort study “Needs, health service use, costs and health-related quality of life in a large sample of oldest-old primary care patients (85 +)” (AgeQualiDe). *n* = 648 individuals were included in the analytical sample. At FU wave 7, mean age was 88.8 years (SD: 2.9 years, from 85 to 99 years). Social support was quantified using the Lubben Social Network Scale (6-item version). Health-related quality of life was assessed using the EQ-5D-3L including problems in five health dimensions, and its visual analogue scale (EQ VAS). It was adjusted for several covariates in conditional logistic and linear fixed effects regressions.

**Results:**

Intraindividual decreases in social support were associated with an increased likelihood of developing problems in ‘self-care’, ‘usual activities’, ‘pain/discomfort’ and ‘anxiety/depression’ (within individuals over time). In contrast, intraindividual changes in social support were not associated with intraindividual changes in the EQ VAS score.

**Conclusion:**

Findings indicate a longitudinal intraindividual association between social support and problems, but only in some health dimensions. Further research in this area based on longitudinal studies among the oldest old (from different countries) is required.

**Supplementary Information:**

The online version contains supplementary material available at 10.1007/s11136-021-03070-2.

## Introduction

It is estimated that particularly the number of individuals in oldest age (85 years and older [1, 2]) will considerably increase in the next decades [3]. In very old age (85 years and older), several critical life events occur such as the death of the spouse, relatives or friends. Moreover, it usually becomes increasingly difficult to meet family members and friends, e.g. due to mobility impairments. Furthermore, factors such as obesity, falls or income poverty can lead to decreased social support or social isolation [4–7]. Moreover, there is a general risk of decline in social support due to the dissolution of traditional family networks [8]. Decreased social support in turn is associated with harmful consequences such as cognitive decline [9], or morbidity and mortality [10, 11].

To date, numerous studies have examined the association between social support and health-related quality of life (HRQoL) (e.g. [12–14]). Most existing studies focused on individuals in old age and mainly showed an association between decreased social support and reduced HRQoL. For instance, this has been shown by both cross-sectional (e.g., [15, 16]) and longitudinal studies (e.g. [17, 18]). However, thus far, there are no studies in which the intraindividual association between social support and HRQoL *exclusively* among the *oldest old* is examined. Therefore, our purpose was to examine the intraindividual association between social support and HRQoL among the oldest old using a longitudinal approach.

## Materials and methods

### Sample

In this study, data were taken from follow-up (FU) waves 7 (year 2014/2015) to 9 (year 2016/2017) from the study on “Needs, health service use, costs and health-related quality of life in a large sample of oldest-old primary care patients (85 +)” (AgeQualiDe). The time span between each wave was ten months. The AgeQualiDe study covered primary care patients 85 years and above at FU wave 7. It took place in six rather large cities in Germany (namely, Bonn, Düsseldorf, Hamburg, Leipzig, Mannheim and Munich).

It should be noted that the AgeQualiDe study is an extension and continuation of the “German Study on Ageing, Cognition and Dementia in Primary Care Patients” (AgeCoDe) which began in 2003/2004. This means that AgeCoDe refers to baseline to FU wave 6 and AgeQualiDe refers to FU wave 7 to FU wave 9.

At baseline, the participants were recruited by means of offices of General Practitioners (GP). In each city, 19 to 29 GPs participated in the recruitment process. In sum, 138 GPs were involved. Inclusion criteria were as follows: 75 years and over, free of dementia, ≥ one visit to the GP in the preceding twelve months. In contrast, they were excluded if one or more of these conditions were fulfilled: poor German language skills, GP consultation by home visits only, residence in a nursing home, severe illness the GP would deem fatal within 3 months, deafness, blindness, lack of ability to provide informed consent, and being an irregular patient of the participating practice.

In sum, 6,619 patients were invited to participate in the baseline assessment. Thereof, 3,327 participants took part at the baseline assessment (1,775 individuals refused participation and 1,517 individuals could not be contacted). At baseline, some selection bias was present [19]. Key reasons for leaving the study in later waves were death and refusal. For example, from baseline to FU wave 3, 712 individuals refused participation and 508 individuals died, whereas other reasons (*n* = 133) did not play a main role. This pattern remained similar in the subsequent waves—with refused participation and particularly death as main drivers of attrition in the AgeQualiDe waves (e.g., FU wave 7: 46 individuals refused participation and 136 individuals died; FU wave 8: 17 individuals refused participation and 78 individuals died; FU wave 9: 18 individuals refused participation and 92 individuals died). Additional details are given elsewhere [20]*.*

**Fig. 1:**
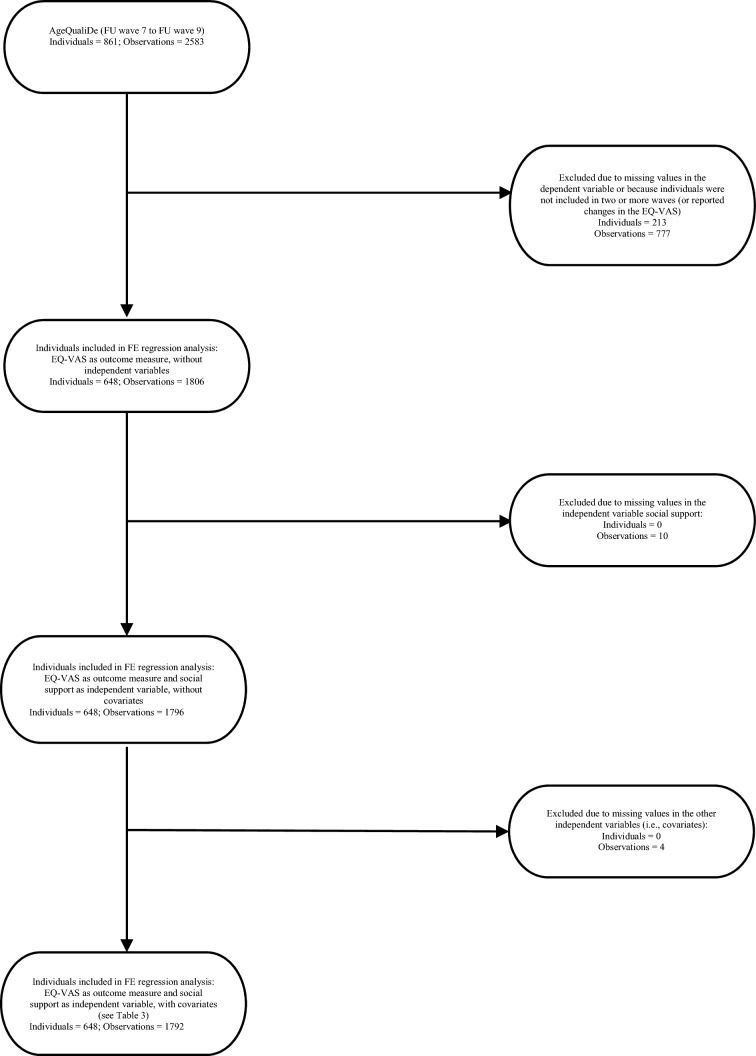
Flow Chart (Individuals included in FE regression analysis)

In our analytical sample (linear FE regressions, see the corresponding regression table), 648 individuals were included (please see Fig. 1 for further details). Our analytical sample solely includes individuals with changes in social support and HRQoL from FU wave 7 to FU wave 9 (additional details are given in the statistical analysis section).

Prior to participation, written informed consent was given by the individuals. The AgeCoDe and the AgeQualiDe-study have been approved by the ethics committees of all participating study centers, and comply with the ethical standards of the Declaration of Helsinki.

### Outcome measure: health-related quality of life

HRQoL was quantified using the established and widely used EQ-5D-3L [21] questionnaire which consists of five items. These items refer to problems in the dimensions ‘mobility’, ‘self-care’, ‘usual activities’, ‘pain/discomfort’, and ‘anxiety/depression’ [21] (in each case: no problems, moderate problems, or extreme problems/unable to can be chosen). Due to data sparseness, these five outcome measures were dichotomized (0 = absence of problems in the respective dimension; 1 = presence of problems in the respective dimension). Furthermore, HRQoL was quantified using the visual analogue scale (EQ VAS), ranging from 0 (worst imaginable health status) to 100 (best imaginable health status).

### Independent variables

Our key time-varying independent variable was social support. It was quantified using the Lubben Social Network Scale (LSNS; 6-item version) which has favorable psychometric characteristics [22]. For example, items are: “How many of your friends do you see or hear from at least once a month?” or “How many relatives do you feel at ease with that you can talk about private matters?” [in each case: 0 = none, 1 = one, 2 = two, 3 = three or four, 4 = five thru eight, and 5 = nine or more]. Each item is equally weighted and this scale ranges from 0 to 30, with higher values corresponding to higher social support/social network. In our current study, Cronbach’s alpha was 0.72 in wave 7 (wave 8: 0.76, wave 9: 0.74).

With regard to time-varying covariates, it was adjusted for age and marital status (married; divorced; single; widowed). Furthermore, it was adjusted for self-rated visual and hearing impairments as well as dementia (using the Global Deterioration Scale [23] with a cut-off of ≥ 4). The Global Deterioration Scale ranges from 1 to 7, with higher values corresponding to more severe cognitive impairment (for example, stage 1 is defined as absence of complaints or objective impairment; stage 2 is defined as the presence of subjective cognitive complaints without objective impairment; stages to 3 to 7 defined as increasing degrees of objective impairments).

Self-rated visual and hearing impairments each were assessed using a 4-point scale (no impairment; mild; severe; profound). Due to the number of cases, both scales were dichotomized (no impairment vs. mild/severe/profound).

With regard to time-constant covariates (i.e., factors that do not change within old individuals over time), we used sex and education (Comparative Analysis of Social Mobility in Industrial Nations (CASMIN) [24] classification, distinguishing between primary, secondary and tertiary education) for descriptive purposes.

### Statistical analysis and sensitivity analyses

Sample characteristics for our analytical sample stratified by wave were displayed. As suggested by other studies (e.g., [25, 26]) focusing on the determinants of well-being outcomes longitudinally, FE regressions were used to explore the intraindividual association between social support and HRQoL. As outcome measures, we first used problems in EQ-5D dimensions (in each case: no problems vs. moderate/extreme problems combined into one category). In these cases, conditional FE logistic regressions were used. Second, the EQ-VAS served as additional outcome measure. In this case, linear FE regressions were applied.

Unlike other regression techniques such as pooled OLS regressions or random effects (RE) regressions, FE regressions provide consistent estimates even when time-constant unobserved (e.g. genetic factors) and observed factors exist which are systematically associated with the explanatory variables [27] (when the strict exogeneity assumption holds). Our choice to use FE regressions (vs. RE regressions) was also supported by Hausman-tests [28] (e.g., with EQ-VAS as outcome measure: Sargan-Hansen statistic was 30.00, *p* < 0.001).

A key feature of FE regressions is that it exclusively exploits variations within individuals over time [27]. Due to this analytical choice, we can only examine whether intraindividual changes in social support are associated with intraindividual changes in HRQoL over time. Therefore, our findings can be interpreted as an average treatment effect on the treated (ATET) – when using the terminology from the counterfactual literature. For further details, please see Brüderl and Ludwig [29].

In wave 7, the proportion of missing values in the explanatory variables ranged from 0% to 1.2% (wave 8: 0% to 2.2%; wave 9: 0% to 1.0%). The proportion of missing values in the dependent variables ranged from 0.9% to 1.6% in wave 7 (wave 8: 0.3% to 1%; wave 9: 0.2% to 1.8%). In additional analysis, full information maximum likelihood (FIML) [30] was used to address the issue of missing data (in the case of linear FE regressions where such a FIML approach is available).

Because panel attrition can affect our results, a sensitivity analysis was conducted which was suggested by Brüderl and Ludwig [31]. In this analysis, only those individuals were included who continuously replied to the outcome measures from wave 7 to wave 9. If results remained very similar, then it is quite unlikely that panel attrition biases the estimates [31].

In a further sensitivity analysis, chronic conditions (recorded by the GP; count score from 0 to 35, for example including diabetes, asthma, back pain or Parkinson’s disease) were added as time-varying covariate. It was only included in sensitivity analysis since various GPs were also retired in the AgeQualiDe waves and therefore dropped out from this study.

Additionally, in another sensitivity analysis, the LSNS total score was replaced by the Family subscale (sum of the first three items of the LSNS-6) and the Friend Subscale (sum of the last three items of the LSNS-6). Moreover, in a last sensitivity analysis, the LSNS total score was replaced by social isolation (absence of social isolation if LSNS-6 ≥ 12; presence of social isolation if LSNS-6 < 12).

The statistical significance was defined as p value of ≤ 0.05. Marginal significance was defined as a p value that ranged from 0.05 to 0.10. Statistical analyses were performed using Stata 16.0 (Stata Corp., College Station, Texas). The Stata syntax was added as Supplementary file 2.

It should be noted that our understanding of FE regressions is, for example, in line with with the understanding of Gunasekara et al. (field of epidemiology) [32], Brüderl and Ludwig (field of sociology) [29] or Cameron and Trivedi (field of economics) [27]. Therefore, FE regressions only use variation within individuals and hence are not affected by confounding from time-invariant factors (both, measured and unmeasured) (for example, we used, among other things, the ‘xtreg’ command in Stata with the ‘fe’ option). For further details regarding the terminology please see Gunasekara et al. [32].

Prior to our FE regression analysis, we also checked whether there is sufficient variation within individuals over time in our key independent variable (to get reliable estimates) using the ‘xttrans’ and the ‘xttab’ command in Stata. Since there is sufficient intraindividual variation over time, we are confident that the estimates are reliable.

## Results

### Sample characteristics

Sample characteristics for our analytical sample (with *n* = 648 individuals; stratified by time) are depicted in Table 1. At FU wave 7, mean age was 88.8 years (SD: 2.9 years), from 85 to 99 years. Most of the individuals were female (67.8%) and had a primary education (56.4%). The mean EQ-VAS score was 63.6 (SD: 18.4). Common problems include ‘pain/discomfort’ (65.6%), ‘mobility’ (60.9%), and to a lesser extent ‘usual activities’ (40.3%), ‘self-care’ (25.6%) as well as ‘anxiety/depression’ (19.6%). Further details are given in Table 1.

**Table 1:** Sample characteristics for the analytical sample (*n* = 648 individuals) stratified by time

Variables	Categories	FU wave 7 (*n* = 640)	FU wave 8 (*n* = 627)	FU wave 9 (*n* = 525)
		M (SD) / n (%)	M (SD) / n (%)	M (SD) / n (%)
Age		88.8 (2.9)	89.6 (2.8)	90.4 (2.7)
Sex	Female	434 (67.8%)	426 (67.9%)	362 (68.9)
	Male	206 (32.2%)	201 (32.1%)	163 (31.1%)
Educational level (CASMIN classification)	Primary	361 (56.4%)	351 (56.0%)	297 (56.6%)
	Secondary	188 (29.4%)	190 (30.3%)	153 (29.1%)
	Tertiary	91 (14.2%)	86 (13.7%)	75 (14.3%)
Marital status	Single/Divorced/Widowed	479 (74.8%)	475 (75.8%)	405 (77.1%)
	Married	161 (25.2%)	152 (24.2%)	120 (22.9%)
Social support (Lubben Social Network Scale; from 0 to 30; high values reflect high social support/social network)	Total score	14.0 (5.3); ranging from 1 to 29	13.7 (5.5); ranging from 0 to 30	13.4 (5.1); ranging from to 0 to 30
	Family Subscale	8.1 (3.3); ranging from 0 to 15	8.0 (3.3); ranging from 0 to 15	7.9 (3.2); ranging from 0 to 15
	Friend Subscale	5.9 (3.7); ranging from 0 to 15	5.7 (3.9); ranging from 0 to 15	5.5 (3.5); ranging from 0 to 15
	Presence of social isolation	197 (30.8%)	225 (35.9%)	193 (36.8%)
Dementia (Global Deterioration Scale; ≥ 4)	Presence of dementia	39 (6.1%)	58 (9.3)	45 (8.6%)
Visual impairment	Mild/Severe/profound	183 (28.6%)	191 (30.5%)	183 (34.9%)
Hearing impairment	Mild/Severe/profound	365 (57.0%)	359 (57.3%)	344 (65.5%)
Health-related quality of life	EQ-VAS	63.6 (18.4)	62.9 (19.0)	61.9 (18.8)
	Presence of problems: Mobility	390 (60.9%)	417 (66.5%)	372 (70.9%)
	Presence of problems: Self-care	164 (25.6%)	191 (30.5%)	178 (33.9%)
	Presence of problems: Usual activities	258 (40.3%)	298 (47.5%)	264 (50.3%)
	Presence of problems: Pain/discomfort	420 (65.6%)	411 (65.6%)	374 (71.2%)
	Presence of problems: Anxiety/depression	125 (19.6%)	162 (25.8%)	143 (27.2%)

### Regression analysis

Findings of conditional FE logistic regression analysis are shown in Table 2 (with problems in the EQ-5D dimensions as outcome measures) and findings of linear FE regression analysis are shown in Table 3 (with EQ-VAS as outcome measure). In the first case, odds ratios (OR) were reported and in the second case, beta-coefficients were reported. For non-continuous variables—e.g., the nominal time-varying covariate dementia -, the coefficients in Table 2 and Table 3 refer to the association between the onset of dementia and the outcome measures.

**Table 2:** Correlates of health-related quality of life (problems in the EQ-5D dimensions: 0 = absence of problems in the respective dimension; 1 = presence of problems in the respective dimensions). Findings of conditional FE logistic regressions

Independent variables	Problems: Mobility	Problems: Self-care	Problems: Usual activities	Problems: Pain/discomfort	Problems: Anxiety/depression
Social support (Lubben Social Network Scale)	0.96 (0.90—1.03)	0.91* (0.85—0.98)	0.95 + (0.89—1.01)	0.93* (0.88—0.99)	0.94 + (0.89—1.00)
Age	1.82*** (1.44—2.31)	1.84*** (1.40—2.41)	1.66*** (1.34—2.06)	1.23* (1.01—1.51)	1.71*** (1.37—2.15)
Married (Ref.: single/divorced/widowed)	0.52 (0.08—3.42)	0.71 (0.10—5.09)	1.44 (0.24—8.84)	0.77 (0.15—3.85)	3.00 (0.38—23.70)
Dementia (Global Deterioration Scale ≥ 4)	0.56 (0.13—2.39)	0.96 (0.26—3.61)	2.05 (0.39—10.89)	0.54 (0.16—1.86)	0.39 (0.11—1.40)
Visual impairment (Ref.: absence of visual impairment)	3.87* (1.26—11.92)	1.67 (0.79—3.52)	1.75 (0.89—3.42)	0.50* (0.27—0.95)	1.02 (0.53—1.96)
Hearing impairment (Ref.: absence of hearing impairment)	1.48 (0.70—3.11)	1.48 (0.67—3.26)	2.04* (1.01—4.14)	0.85 (0.43—1.67)	1.04 (0.48—2.28)
Observations	511	435	602	614	537
Number of Individuals	178	155	211	216	189
Pseudo R^2^	.12	.14	.10	.04	.08

Odds ratios are presented; 95%-CI in parentheses

^*^p < 0.05

^**^p < 0.01

^***^p < 0.001

+ *p *< 0.10

**Table 3:** Correlates of health-related quality of life (EQ-VAS, ranging from 0 (worst) to 100 (best)). Findings of linear FE regressions

Independent variables	EQ-VAS
Social support (Lubben Social Network Scale)	0.02
	(0.14)
Age	−0.97*
	(0.46)
Married (Ref.: single/divorced/widowed)	−2.97
	(3.28)
Dementia (Global Deterioration Scale ≥ 4)	−7.20*
	(3.11)
Visual impairment (Ref.: absence of visual impairment)	−1.40
	(1.71)
Hearing impairment (Ref.: absence of hearing impairment)	−3.14*
	(1.50)
Constant	153.38***
	(40.86)
Observations	1792
Individuals	648
R^2^	0.02

Unstandardized beta-coefficients are reported; cluster-robust standard errors in parentheses

^*^p < 0.05

^**^p < 0.01

^***^p < 0.001

+ *p *< 0.10

Adjusting for several time-varying covariates, conditional FE logistic regressions revealed that intraindividual decreases in social support were associated with an increased likelihood of developing problems in ‘self-care’ (OR = 0.91, *p* < 0.05), ‘usual activities’ (OR = 0.95, *p* < 0.10), ‘pain/discomfort’ (OR = 0.93, *p* < 0.05) and ‘anxiety/depression’ (OR = 0.94, *p* < 0.10) within individuals over time. Beyond that, only increasing age (intraindividual) was consistently associated with an increased likelihood of problems in all five dimensions within individuals over time.

Linear FE regressions did not show an association between intraindividual changes in social support and intraindividual changes in the EQ-VAS score. Intraindividual decreases in the EQ-VAS score were associated with intraindividual increases in age (β = −0.97, *p* < 0.05), the onset of dementia within individuals over time (β = −7.20, *p* < 0.05), and the onset of hearing impairment within individuals over time (β = −3.14, *p* < 0.05).

In additional analysis, full information maximum likelihood (FIML) was used to tackle missing data in linear FE regressions (please see Supplementary file 1: Supplementary Table 1). Similarly, in this model, intraindividual decreases in the EQ-VAS score were associated with the onset of dementia within individuals over time (β = −6.71, *p* < 0.05), and the onset of hearing impairment within individuals over time (β = −3.66, *p* < 0.05), whereas the association between intraindividual increases in age and intraindividual decreases in the EQ-VAS score vanished.

Moreover, in another sensitivity analysis, we restricted FE regressions to those individuals who continuously replied to the outcome measures from wave 7 to wave 9 (please see Supplementary file 1: Supplementary Table 2). While the marginal significant associations between intraindividual decreases in social support and intraindividual increases in the likelihood of developing problems in ‘usual activities’ within individuals over time (OR = 0.95, *p* = 0.15) and ‘anxiety/depression’ within individuals over time (OR = 0.97, *p* = 0.38) vanished, the significant associations between intraindividual decreases in social support and increases in the likelihood of developing problems in ‘self-care’ within individuals over time (OR = 0.91, *p* < 0.05) and ‘pain/discomfort’ within individuals over time remained nearly the same (OR = 0.92, *p* < 0.01).

In further sensitivity analysis, chronic conditions were added as a time-varying covariate to our main model (please see Supplementary file 1: Supplementary Table 3). Some differences (compared to our main model) are worth noting: While a marginal significant association between intraindividual decreases in social support and increases in the likelihood of developing problems in ‘mobility’ within individuals over time (OR: 0.93, *p* = 0.06) appeared, the marginal significant associations with ‘usual activities’ within individuals over time (OR: 0.96, *p* = 0.24) and ‘anxiety/depression’ within individuals over time (OR = 0.95, *p* = 0.14) disappeared. Furthermore, while the association between intraindividual decreases in social support and increases in the likelihood of developing problems in ‘self-care’ within individuals over time (OR = 0.93, *p* = 0.11) disappeared, the association with ‘pain/discomfort’ within individuals over time remained nearly the same (OR = 0.91, *p* < 0.01).

In further sensitivity analysis, the LSNS total score was replaced by the Family subscale and the Friend Subscale (please see Supplementary file 1: Supplementary Table 4). While intraindividual decreases in the family subscale (i.e., lower support from family) were associated with increases in the likelihood of developing problems in ‘self-care’ within individuals over time (OR: 0.88, *p* < 0.10) and ‘usual activities’ within individuals over time (OR: 0.85, *p* < 0.01), intraindividual decreases in the friend subscale (i.e., lower support from friends) were associated with increases in the likelihood of developing problems in ‘pain/discomfort’ within individuals over time (OR: 0.93, *p* < 0.10).

In our last sensitivity analyses, the LSNS total score was replaced by social isolation (dichotomized LSNS-6; please see Supplementary File 1: Supplementary Table 5). Our key findings remained similar. More precisely, while the marginal significant association between the presence of social isolation within individuals over time and increases in the likelihood of developing problems in ‘usual activities’ within individuals over time (OR: 1.04, *p* = 0.89) disappeared, the presence of social isolation within individuals over time was still associated with intraindividual increases in the likelihood of developing problems in ‘self-care’ (OR: 1.92, *p* < 0.05), ‘pain/discomfort’ (OR: 2.01, *p* < 0.05) and ‘anxiety/depression’ (OR: 1.60, *p* < 0.10).

## Discussion

The objective of this study was to examine the association between social support and HRQoL among the oldest old longitudinally. Adjusting for various covariates, conditional logistic FE regressions showed that intraindividual decreases in social support were associated with an increased likelihood of developing problems in ‘self-care’, ‘usual activities’ (marginal significant), ‘pain/discomfort’ and ‘anxiety/depression’ (marginal significant) (within individuals over time). In contrast, linear FE regressions showed that intraindividual changes in social support were not associated with intraindividual changes in the EQ VAS score.

Our findings build upon prior knowledge by showing that intraindividual decreases in social support are associated with an increased likelihood of problems in several health dimensions (within individuals) longitudinally solely focusing on the oldest old. Our findings appear to be plausible because it has been shown that being embedded in social networks is a protective factor against stress and illnesses [33]. This means that strong social ties may act as a buffer (e.g., against pain [34]) – which is clearly in accordance with the buffering hypothesis of Cohen and Wills [33]. Our findings are difficult to compare with previous studies due to differences in age bracket, analytical approach and the tools used to assess social support and HRQoL. For example, previous studies mainly focused on self-rated health as outcome measure (e.g., [16, 35]).

However, our study did not find an association between intraindividual changes in social support and intraindividual changes in the EQ VAS score. A possible mechanism may be that strong social support may result in additional stress which can ultimately reduce HRQoL [36]. For example, increased social support within individuals over time among the oldest old may at least partly reflect a need for care, combined with guilt as it may seem not possible to repay the support [4]. This need for care may reflect a rather unidirectional relationship to friends and acquaintances because these individuals with increased social support may feel guilty and may feel unable to repay the support they receive from friends or relatives [4]. Thus, this dissatisfaction with the strong social support may produce stress, which can lead to decreased HRQoL. This process, which may be present in some individuals, may counterbalance the positive effects of social support, which may be present in other individuals. However, future research is required to clarify this issue.

Moreover, another possible explanation may be that – in contrast to problems in health dimensions where HRQoL is indirectly measured—the EQ VAS score directly refers to HRQoL. For example, individuals may have coped with losses (such as reductions in social support due to death of friends or relatives) [37] which in turn does not affect their assessment of HRQoL. Additionally, our findings suggest that intraindividual changes in health-related factors (i.e., dementia and hearing impairment) can contribute to intraindividual changes in the EQ VAS score.

Our study has some strengths and limitations. This is the first longitudinal study investigating the within-association between social support and health-related quality of life exclusively among the oldest old. We used data from a multicenter prospective cohort study (AgeCoDe/AgeQualiDe). Established and widely applied tools were used to quantify our main independent variable (social support) and our dependent variable (HRQoL). The problem of unobserved heterogeneity was diminished using FE regressions. When interpreting our FE results, it should be repeated that our results refer to an ATET. However, as argued by Brüderl and Ludwig [29], this is not a shortcoming of FE estimates because it simply reflects the facts that a certain proportion of the real world population does not change social support. It should be noted that our sensitivity analyses suggested that panel attrition may slightly bias our estimates. Moreover, adding chronic conditions to our model led to slightly different findings which may be partly explained by the loss of observations (since several GPs did not fill out the questionnaires anymore).

While the baseline assessment of the AgeCoDe/AgeQualiDe study was a nearly representative sample of the older population residing in Germany [38], it is worth emphasizing that some sample selection bias and panel attrition (please see above) exist in this study [19, 39]. This may bias our analytical sample towards more healthy participants (please see [40]). Moreover, further covariates (e.g., personality factors such as extraversion) could be included in future studies. Additionally, the possibility of a reverse causality (endogeneity) cannot be entirely dismissed (e.g., problems with depression leads to changes in social support [41]) – and should be further investigated.

## Conclusion

Findings indicate a longitudinal intraindividual association between social support and problems in several health dimensions. Further research in this area based on longitudinal studies among the oldest old is required.

## Supplementary Information

Below is the link to the electronic supplementary material.

Supplementary file1 (PDF 252 KB)

Supplementary file2 (TXT 12 KB)

## Data Availability

Due to ethical restrictions involving patient data, underlying data are available upon request from the Working Group Medical Statistics and IT-Infrastructure. Contact information: Birgitt Wiese, wiese.birgitt@mh-hannover.de.

## Code availability

Please see Supplementary file 2.
