# Supplementary material for: Social support and health-related quality of life among the oldest old — longitudinal evidence from the multicenter prospective AgeCoDe-AgeQualiDe study
Source: Qual Life Res. 2021 Dec 22;31(6):1667–76. doi: 10.1007/s11136-021-03070-2 (PMC9098616; doi:10.1007/s11136-021-03070-2)
Supplement: Supplementary file 1 — Supplementary file1 (PDF 252 KB) [file 11136_2021_3070_MOESM1_ESM.pdf]

Supplementary File 1.

Reference:

Authors: André Hajek, Christian Brettschneider, Tina Mallon, Hanna Kaduszkiewicz, Anke Oey, Birgitt Wiese, Siegfried Weyerer, Jochen Werle, Michael Pentzek, Angela Fuchs, Ines Conrad, Melanie Luppa, Dagmar Weeg, Edelgard Mösch, Luca Kleineidam, Michael Wagner, Martin Scherer, Wolfgang Maier, Steffi G. Riedel-Heller*, Hans-Helmut König*

* equal contributors

Title: Social support and health-related quality of life among the oldest old – Longitudinal evidence from the multicenter prospective AgeCoDe-AgeQualiDe study

Statement of Ethics

Prior to participation, written informed consent was given by all participants. The AgeCoDe and the AgeQualiDe-study have been approved by the ethics committees of all participating study centers and comply with the ethical standards of the Declaration of Helsinki.

- Ethics Commission of the Medical Association Hamburg (reference number: MC-390/13)
- Ethics Committee of the Medical Faculty of the Rheinische Friedrich-Wilhelms-University of Bonn (reference number: 369/13)
- Medical Ethics Commission II of the Medical Faculty Mannheim/Heidelberg University (reference number: 2013-662 N-MA)
- Ethics Committee of the Faculty of Medicine of the University of Leipzig (reference number: 309/2007; 333-13-18112013)
- Ethical Committee of the Medical Faculty of the Heinrich-Heine-University Düsseldorf (reference number: 2999)
- Ethics Committee of the Faculty of Medicine of the Technical University of Munich (reference number: 713/02 E)

Supplementary Table 1. Correlates of health-related quality of life (EQ-VAS, ranging from 0 (worst) to 100 (best)). Findings of linear FE regressions (with full information maximum likelihood to address missing values)

Independent variables	EQ-VAS
Social support (Lubben Social Network Scale)	0.04
	(0.14)
Age	-0.93
	(1.29)
Married (Ref.: single/divorced/widowed)	-2.55
	(3.55)
Dementia (Global Deterioration Scale ≥ 4)	-6.71*
	(2.69)
Visual impairment (Ref.: absence of visual impairment)	-1.34
	(1.39)
Hearing impairment (Ref.: absence of hearing impairment)	-3.66*
	(1.47)

Unstandardized beta-coefficients are reported; cluster-robust standard errors in parentheses; *** p<0.001, ** p<0.01, * p<0.05, + p<0.10; 861 individuals (three periods).

Supplementary Table 2. Correlates of health-related quality of life. Findings of conditional FE logistic regressions (problems) and findings of linear FE regressions (EQ-VAS) - only including those individuals who continuously replied to the outcome measures from wave 7 to wave 9

Independent variables	Problems: Mobility	Problems: Self-care	Problems: Usual activities	Problems: Pain/discomfort	Problems: Anxiety/depression	EQ-VAS
Social support (Lubben Social Network Scale)	0.95	0.91*	0.95	0.92**	0.97	0.05
	(0.89 - 1.02)	(0.84 - 0.99)	(0.89 - 1.02)	(0.86 - 0.98)	(0.91 - 1.04)	(0.14)
Age	1.76***	1.92***	1.67***	1.21+	1.64***	-0.96*
	(1.38 - 2.25)	(1.44 - 2.57)	(1.34 - 2.08)	(0.98 - 1.50)	(1.30 - 2.08)	(0.46)
Married (Ref.: single/divorced/widowed)	0.51	0.99	1.00	0.99	2.91	-1.29
	(0.08 - 3.35)	(0.11 - 8.54)	(0.09 - 11.39)	(0.17 - 5.64)	(0.38 - 22.17)	(3.28)
Dementia (Global Deterioration Scale ≥ 4)	0.70	1.08	1.67	0.65	0.63	-7.80*
	(0.14 - 3.39)	(0.23 - 5.02)	(0.30 - 9.24)	(0.18 - 2.34)	(0.15 - 2.66)	(3.40)
Visual impairment (Ref.: absence of visual impairment)	4.30*	1.98	1.60	0.49*	0.84	-1.55
	(1.19 - 15.47)	(0.80 - 4.87)	(0.78 - 3.28)	(0.25 - 0.93)	(0.42 - 1.69)	(1.83)
Hearing impairment (Ref.: absence of hearing impairment)	1.42	1.30	2.34*	0.91	0.97	-3.51*
	(0.67 - 3.02)	(0.55 - 3.09)	(1.11 - 4.93)	(0.45 - 1.81)	(0.40 - 2.35)	(1.61)
Constant						151.70***
						(41.03)
Observations	469	379	544	556	477	1,516
Number of Individuals	157	127	182	187	159	510
(Pseudo) R^2	0.12	0.16	0.10	0.04	0.06	0.02

In case of conditional FE logistic regressions: Odds ratios are presented; 95%-CI in parentheses.
In case of linear FE regressions: Unstandardized beta-coefficients are reported; cluster-robust standard errors in parentheses.
*** p<0.001, ** p<0.01, * p<0.05, + p<0.10

Supplementary Table 3. Correlates of health-related quality of life. Findings of conditional FE logistic regressions (problems) and findings of linear FE regressions (EQ-VAS) – with chronic conditions as additional time-varying covariate

Independent variables	Problems: Mobility	Problems: Self-care	Problems: Usual activities	Problems: Pain/discomfort	Problems: Anxiety/depression	EQ-VAS
Social support (Lubben Social Network Scale)	0.93+	0.93	0.96	0.91**	0.95	0.03
	(0.86 - 1.00)	(0.85 - 1.02)	(0.89 - 1.03)	(0.85 - 0.98)	(0.88 - 1.02)	(0.16)
Age	1.92***	2.15***	1.53***	1.23+	1.85***	-0.81
	(1.43 - 2.59)	(1.50 - 3.06)	(1.19 - 1.97)	(0.97 - 1.56)	(1.39 - 2.47)	(0.54)
Married (Ref.: single/divorced/widowed)	0.57	0.97	2.29	0.78	2.19	-4.87
	(0.08 - 4.03)	(0.09 - 10.26)	(0.33 - 15.79)	(0.16 - 3.89)	(0.19 - 25.38)	(3.26)
Dementia (Global Deterioration Scale ≥ 4)	0.31	0.76	1.97	0.30	0.29	-5.20
	(0.04 - 2.38)	(0.16 - 3.65)	(0.34 - 11.61)	(0.05 - 1.70)	(0.06 - 1.44)	(3.51)
Visual impairment (Ref.: absence of visual impairment)	4.20*	1.18	2.62*	0.56	0.53	-2.89
	(1.13 - 15.61)	(0.43 - 3.19)	(1.02 - 6.69)	(0.27 - 1.14)	(0.22 - 1.26)	(2.12)
Hearing impairment (Ref.: absence of hearing impairment)	2.08	1.76	1.82	0.77	1.23	-2.73
	(0.77 - 5.67)	(0.70 - 4.38)	(0.79 - 4.19)	(0.35 - 1.69)	(0.52 - 2.89)	(1.76)
Chronic conditions (count score)	1.32**	1.07	1.01	1.03	1.13+	-0.58*
	(1.09 - 1.60)	(0.91 - 1.26)	(0.88 - 1.17)	(0.91 - 1.16)	(0.98 - 1.30)	(0.29)
Constant						143.93**
						(47.93)
Observations	367	301	428	455	368	1,302
Number of Individuals	132	111	156	162	134	487
(Pseudo) R^2	0.20	0.17	0.08	0.05	0.12	0.03

In case of conditional FE logistic regressions: Odds ratios are presented; 95%-CI in parentheses.
In case of linear FE regressions: Unstandardized beta-coefficients are reported; cluster-robust standard errors in parentheses.
*** p<0.001, ** p<0.01, * p<0.05, + p<0.10

Supplementary Table 4. Correlates of health-related quality of life. Findings of conditional FE logistic regressions (problems) and findings of linear FE regressions (EQ-VAS) – with family subscale and friend subscale

Independent variables	Problems: Mobility	Problems: Self-care	Problems: Usual activities	Problems: Pain/discomfort	Problems: Anxiety/depression	EQ-VAS
Family Subscale (Lubben Social Network Scale)	0.93	0.88+	0.85**	0.93	0.94	0.05
	(0.82 - 1.05)	(0.78 - 1.00)	(0.75 - 0.95)	(0.84 - 1.04)	(0.84 - 1.05)	(0.24)
Friend Subscale (Lubben Social Network Scale)	0.98	0.93	1.01	0.93+	0.95	0.01
	(0.90 - 1.07)	(0.85 - 1.03)	(0.93 - 1.10)	(0.86 - 1.00)	(0.87 - 1.03)	(0.18)
Age	1.84***	1.86***	1.70***	1.23*	1.72***	-0.98*
	(1.45 - 2.34)	(1.41 - 2.46)	(1.37 - 2.11)	(1.01 - 1.51)	(1.37 - 2.15)	(0.46)
Married (Ref.: single/divorced/widowed)	0.56	0.72	1.54	0.77	2.99	-3.02
	(0.08 - 3.83)	(0.10 - 5.25)	(0.25 - 9.56)	(0.15 - 3.85)	(0.38 - 23.69)	(3.31)
Dementia (Global Deterioration Scale ≥ 4)	0.55	0.98	2.30	0.54	0.38	-7.19*
	(0.13 - 2.33)	(0.26 - 3.66)	(0.42 - 12.51)	(0.16 - 1.86)	(0.11 - 1.39)	(3.11)
Visual impairment (Ref.: absence of visual impairment)	4.07*	1.63	1.73	0.51*	1.02	-1.39
	(1.30 - 12.70)	(0.77 - 3.44)	(0.88 - 3.40)	(0.27 - 0.96)	(0.53 - 1.96)	(1.71)
Hearing impairment (Ref.: absence of hearing impairment)	1.50	1.44	2.03*	0.85	1.04	-3.14*
	(0.71 - 3.15)	(0.65 - 3.18)	(1.00 - 4.12)	(0.43 - 1.67)	(0.48 - 2.28)	(1.50)
Constant						153.60***
						(40.89)
Observations	511	435	602	614	537	1,792
Number of Individuals	178	155	211	216	189	648
(Pseudo) R^2	0.12	0.14	0.11	0.04	0.08	0.02

In case of conditional FE logistic regressions: Odds ratios are presented; 95%-CI in parentheses.
In case of linear FE regressions: Unstandardized beta-coefficients are reported; cluster-robust standard errors in parentheses.
*** p<0.001, ** p<0.01, * p<0.05, + p<0.10

Supplementary Table 5. Correlates of health-related quality of life. Findings of conditional FE logistic regressions (problems) and findings of linear FE regressions (EQ-VAS) – with social isolation

Independent variables	Problems: Mobility	Problems: Self-care	Problems: Usual activities	Problems: Pain/discomfort	Problems: Anxiety/depression	EQ-VAS
Social isolation (Lubben Social Network Scale < 12)	1.05	1.92*	1.04	2.01*	1.60+	0.33
	(0.57 - 1.93)	(1.01 - 3.65)	(0.61 - 1.77)	(1.16 - 3.48)	(0.93 - 2.76)	(1.33)
Age	1.84***	1.87***	1.70***	1.23*	1.72***	-1.00*
	(1.45 - 2.34)	(1.43 - 2.45)	(1.37 - 2.10)	(1.01 - 1.51)	(1.37 - 2.16)	(0.46)
Married (Ref.: single/divorced/widowed)	0.56	0.79	1.46	0.77	3.17	-2.94
	(0.09 - 3.62)	(0.11 - 5.77)	(0.24 - 8.83)	(0.15 - 3.98)	(0.40 - 25.28)	(3.29)
Dementia (Global Deterioration Scale ≥ 4)	0.55	1.16	2.20	0.55	0.39	-7.15*
	(0.13 - 2.33)	(0.30 - 4.52)	(0.42 - 11.65)	(0.16 - 1.95)	(0.11 - 1.40)	(3.13)
Visual impairment (Ref.: absence of visual impairment)	3.99*	1.71	1.71	0.52*	1.06	-1.39
	(1.30 - 12.20)	(0.81 - 3.63)	(0.88 - 3.33)	(0.27 - 0.99)	(0.54 - 2.06)	(1.71)
Hearing impairment (Ref.: absence of hearing impairment)	1.48	1.44	1.90+	0.92	1.10	-3.11*
	(0.70 - 3.13)	(0.66 - 3.14)	(0.94 - 3.82)	(0.47 - 1.81)	(0.50 - 2.39)	(1.50)
Constant						155.55***
						(40.70)
Observations	511	435	602	614	537	1,792
Number of Individuals	178	155	211	216	189	648
(Pseudo) R^2	0.12	0.13	0.09	0.04	0.08	0.02

In case of conditional FE logistic regressions: Odds ratios are presented; 95%-CI in parentheses.
In case of linear FE regressions: Unstandardized beta-coefficients are reported; cluster-robust standard errors in parentheses.
*** p<0.001, ** p<0.01, * p<0.05, + p<0.10

STROBE Statement—Checklist of items that should be included in reports of ***cohort studies***

	Item No	Recommendation	Page No
**Title and abstract**	1	(*a*) Indicate the study's design with a commonly used term in the title or the abstract	1
		(*b*) Provide in the abstract an informative and balanced summary of what was done and what was found	3
**Introduction**			
Background/rationale	2	Explain the scientific background and rationale for the investigation being reported	4
Objectives	3	State specific objectives, including any prespecified hypotheses	4
**Methods**			
Study design	4	Present key elements of study design early in the paper	4-6
Setting	5	Describe the setting, locations, and relevant dates, including periods of recruitment, exposure, follow-up, and data collection	4-6
Participants	6	(*a*) Give the eligibility criteria, and the sources and methods of selection of participants. Describe methods of follow-up	5-6
		(*b*) For matched studies, give matching criteria and number of exposed and unexposed	Not applicable
Variables	7	Clearly define all outcomes, exposures, predictors, potential confounders, and effect modifiers. Give diagnostic criteria, if applicable	6-7
Data sources/ measurement	8*	For each variable of interest, give sources of data and details of methods of assessment (measurement). Describe comparability of assessment methods if there is more than one group	6-7
Bias	9	Describe any efforts to address potential sources of bias	7-9
Study size	10	Explain how the study size was arrived at	5-6
Quantitative variables	11	Explain how quantitative variables were handled in the analyses. If applicable, describe which groupings were chosen and why	6-7
Statistical methods	12	(*a*) Describe all statistical methods, including those used to control for confounding	7-9
		(*b*) Describe any methods used to examine subgroups and interactions	Not applicable
		(*c*) Explain how missing data were addressed	8
		(*d*) If applicable, explain how loss to follow-up was addressed	8
		(*e*) Describe any sensitivity analyses	8-9
**Results**			
Participants	13*	(a) Report numbers of individuals at each stage of study—eg numbers potentially eligible, examined for eligibility, confirmed eligible, included in the study, completing follow-up, and analysed	9-10
		(b) Give reasons for non-participation at each stage	5
		(c) Consider use of a flow diagram	24
Descriptive data	14*	(a) Give characteristics of study participants (eg demographic, clinical,	9-10; 21

		social) and information on exposures and potential confounders	
		(b) Indicate number of participants with missing data for each variable of interest	9
		(c) Summarise follow-up time (eg, average and total amount)	21
Outcome data	15*	Report numbers of outcome events or summary measures over time	10-12

Main results	16	(*a*) Give unadjusted estimates and, if applicable, confounder-adjusted estimates and their precision (eg, 95% confidence interval). Make clear which confounders were adjusted for and why they were included	10-12
		(*b*) Report category boundaries when continuous variables were categorized	21
		(*c*) If relevant, consider translating estimates of relative risk into absolute risk for a meaningful time period	Not applicable
Other analyses	17	Report other analyses done—eg analyses of subgroups and interactions, and sensitivity analyses	11-12
**Discussion**			
Key results	18	Summarise key results with reference to study objectives	12-13
Limitations	19	Discuss limitations of the study, taking into account sources of potential bias or imprecision. Discuss both direction and magnitude of any potential bias	14-15
Interpretation	20	Give a cautious overall interpretation of results considering objectives, limitations, multiplicity of analyses, results from similar studies, and other relevant evidence	12-14
Generalisability	21	Discuss the generalisability (external validity) of the study results	14-15
**Other information**			
Funding	22	Give the source of funding and the role of the funders for the present study and, if applicable, for the original study on which the present article is based	15

*Give information separately for exposed and unexposed groups.

Note: An Explanation and Elaboration article discusses each checklist item and gives methodological background and published examples of transparent reporting. The STROBE checklist is best used in conjunction with this article (freely available on the Web sites of PLoS Medicine at http://www.plosmedicine.org/, Annals of Internal Medicine at http://www.annals.org/, and Epidemiology at http://www.epidem.com/). Information on the STROBE Initiative is available at http://www.strobe-statement.org.
